# Supplementary material for: ‘Off pump’ self-expanding injectable tissue valves (IPVR) versus ‘on pump’ conventional tissue valves (PVR) for replacement of the pulmonary valve: trial protocol for a randomised controlled trial (InVITe trial)
Source: BMJ Open. 2019 Apr 2;9(4):e026221. doi: 10.1136/bmjopen-2018-026221 (PMC6500208; doi:10.1136/bmjopen-2018-026221)
Supplement: Author's manuscript [file bmjopen-2018-026221.draft_revisions.pdf]

BMJ Open is committed to open peer review. As part of this commitment we make the peer review history of every article we publish publicly available.

When an article is published we post the peer reviewers' comments and the authors' responses online. We also post the versions of the paper that were used during peer review. These are the versions that the peer review comments apply to.

The versions of the paper that follow are the versions that were submitted during the peer review process. They are not the versions of record or the final published versions. They should not be cited or distributed as the published version of this manuscript.

BMJ Open is an open access journal and the full, final, typeset and author-corrected version of record of the manuscript is available on our site with no access controls, subscription charges or pay-per-view fees (http://bmjopen.bmj.com).

If you have any questions on BMJ Open's open peer review process please email editorial.bmjopen@bmj.com

BMJ Open

'Off pump' self-expanding injectable tissue valves (IPVR) versus 'on pump' conventional tissue valves (PVR) for replacement of the pulmonary valve: trial protocol for an early phase randomised controlled trial (InVITe trial)

Journal:	*BMJ Open*
Manuscript ID	bmjopen-2018-026221
Article Type:	Protocol
Date Submitted by the Author:	06-Sep-2018

Complete List of Authors:	Heys, Rachael; University of Bristol , Clinical Trials and Evaluation Unit, Bristol Trials Centre, Bristol Medical School<br>Angelini, Gianni; University of Bristol, Bristol Heart Institute<br>Caputo, Massimo; University Hospitals Bristol NHS Foundation Trust, Bristol Royal Hospital for Children<br>Culliford, Lucy; University of Bristol , Clinical Trials and Evaluation Unit, Bristol Trials Centre, Bristol Medical School<br>Pufulete, Maria; University of Bristol, Clinical Trials and Evaluation Unit, Bristol Trials Centre, Bristol Medical School<br>Reeves, Barnaby; University of Bristol, Clinical Trials and Evaluation Unit, Bristol Trials Centre, Bristol Medical School<br>Rogers, Chris; University of Bristol , Clinical Trials and Evaluation Unit, Bristol Trials Centre, Bristol Medical School<br>Stoica, Serban ; University Hospitals Bristol NHS Foundation Trust<br>Parry , Andrew ; University Hospitals Bristol NHS Foundation Trust, Bristol Royal Hospital for Children

Keywords:	Cardiac surgery < SURGERY, Congenital heart disease < CARDIOLOGY, Valvular heart disease < CARDIOLOGY, Protocols & guidelines < HEALTH SERVICES ADMINISTRATION & MANAGEMENT

SCHOLARONE™ Manuscripts

'Off pump' self-expanding injectable tissue valves (IPVR) versus 'on pump' conventional tissue valves (PVR) for replacement of the pulmonary valve: trial protocol for an early phase randomised controlled trial (InVITe trial)

Rachael Heys[1] rachael.heys@bristol.ac.uk

Gianni Angelini[2] g.d.angelini@bristol.ac.uk

Massimo Caputo[3] m.caputo@bristol.ac.uk

Lucy Culliford[1] lucy.culliford@bristol.ac.uk

Maria Pufulete[1] maria.pufulete@bristol.ac.uk

Barnaby C Reeves[1] barney.reeves@bristol.ac.uk

Chris A Rogers[1] chris.rogers@bristol.ac.uk

Serban Stoica[3] serban.stoica@uhbristol.nhs.uk

Andrew Parry[2] andrew.parry@uhbristol.nhs.uk

1. Clinical Trials and Evaluation Unit, Bristol Trials Centre, Bristol Medical School, University of Bristol, Bristol, UK
2. Bristol Heart Institute, University of Bristol, Bristol, UK
3. Bristol Royal Hospital for Children, University Hospitals Bristol, Bristol, UK

Corresponding author:

Mr Andrew Parry, Bristol Royal Hospital for Children, University Hospitals Bristol, Bristol, UK. Email: Andrew.parry@uhbristol.nhs.uk. Tel: 0117 342 8854

Word count: 3208 (excluding title page, abstract, references, figures and tables)

Number of figures: 1

Number of tables: 1

For peer review only

Keywords: Pulmonary valve; congenital; cardiopulmonary bypass (CPB); self-expanding injectable valve; minimally invasive; cardiac surgery; no-react injectable BioPulmonic valve; randomised controlled trial.

For peer review only

ABSTRACT

Introduction: Patients with congenital heart disease often need repeated operations throughout life to replace the pulmonary valve. Valve replacement with 'injectable' self-expanding valves (which is performed 'off pump'; without the use of cardiopulmonary bypass, CPB) may result in quicker recovery and lower risk of major complications than valve replacement with conventional valves (which is performed 'on pump', with the use of CPB).

Methods and analysis: We are conducting a multi-centre, single-blind randomised controlled trial in patients aged between 12 and 80 years with congenital heart disease. We will randomise participants in a 1:1 ratio to receive either 'off pump' injectable pulmonary valve replacement or 'on pump' conventional pulmonary valve replacement. The primary outcome will be the difference between groups with respect to post-surgery blood loss (as measured by chest drain volume) in the first 24 hours. Secondary outcomes will include in-hospital outcomes (intensive care unit stay; inotropic/vasodilator support; chest drain volume in the first 12 hours post-surgery; time of readiness for extubation; blood products used in the first 24 hours post-surgery; time of fitness for discharge); valve and heart function 6 months post-surgery (assessed using cardiovascular magnetic resonance and echocardiography); and health related quality of life 6 weeks and 6 months post-surgery.

Ethics and dissemination: This trial has been approved by the South West – Exeter Research Ethics Committee. Findings will be shared with participating hospitals and disseminated to the academic community through peer reviewed publications and presentation at national and international meetings. Patients will be informed of the results through patient organisations and newsletters to participants.

Trial registration: ISRCTN registry, identifier ISRCTN23538073. Registered on 20/01/2016.

Strengths and limitations

- This trial is the first randomised controlled trial (RCT) to assess the efficacy of injectable tissue valves versus conventional tissue valves for replacement of the pulmonary valve.
- This is a multi-centre RCT, with computer-generated concealed randomisation and participant blinding to minimise bias.
- We are including both children and adults (age range 12-80 years) to ensure the findings will be generalizable to a wide group of patients who need pulmonary valve replacement.
- Although clinicians cannot be blinded to the patient's treatment allocation we will minimise bias by pre-defining all procedures for data collection and applying these to all participants in the same way.
- The primary and many of the secondary endpoints are clinical outcomes, based on objective criteria, which are important to patients, clinical teams, and the NHS.

INTRODUCTION

Background and objectives

Most children born with complex congenital heart disease now reach adulthood. Post-surgery mortality following surgical correction of Tetralogy of Fallot for example, has fallen from 5.5% in 1980, (1) to 0% in 2016.(2) However, as long term survival has improved late sequelae have become evident. Many of the patients' operated on for tetralogy of Fallot and other forms of pulmonary valve disease require multiple operations throughout life to repair or replace poorly functioning pulmonary valves.

The standard operation for pulmonary valve replacement (PVR) involves opening the chest, exposing the heart and using cardiopulmonary bypass (CPB; 'on pump'). CPB is a technique that temporarily takes over the function of the heart and lungs (pumping blood and oxygen through the body) and occasionally involves stopping the heart during surgery. Use of CPB is associated with complications such as excessive bleeding and reperfusion injury in cases where the heart has to be stopped (damage to heart tissue occurs when the blood supply returns to the heart).

For peer review only

Injectable tissue valves (BioPulmonic™, Biointegral Surgical Inc.) have been developed to replace conventional tissue valves for PVR. Injectable tissue valves can be implanted without the use of CPB ('off pump') and with less dissection of the heart. Case reports and small case series (<12 patients)(3-10) have shown that injectable tissue valves are easy to implant and have satisfactory function. Pilot data has shown shorter operating time (2 hours less), and less blood loss (over 400 ml less) and blood product requirement (3 units less) in 6 'off-pump' injectable pulmonary valve replacement (IPVR) patients compared with 7 'on pump' PVR patients. No patient had a paravalvular leak (leak around the replaced valve) or more than mild pulmonary regurgitation at early follow up, suggesting that IPVR is a safe procedure.(11)

There are no randomised controlled trials (RCTs) that have evaluated IPVR versus PVR. We are conducting an RCT to determine whether IPVR leads to less blood loss, faster recovery, lower risk of post-surgery complications and good valve function in patients who need an operation to replace their pulmonary valve.

AIMS AND OBJECTIVES

The main aim of the InVITe trial is to compare IPVR versus PVR in patients that need pulmonary valve replacement. The objectives are to estimate the difference between groups with respect to:

1. Post-surgery blood loss (as measured by chest drain volume) in the first 24 hours (primary outcome);
2. A range of clinical post-surgery outcomes (intensive care unit (ICU) stay; inotropic/vasodilator support; chest drain volume in the first 12 hours; time until ready for extubation; blood products used in the first 24 hours; fitness for discharge (FFD); valve-related complications during follow-up);
3. Heart and valve function assessed using cardiovascular magnetic resonance (CMR) and echocardiography;
4. Health related quality of life (HRQoL).

METHODS

Trial design and population

The InVITe trial is an early phase, multi-centre, RCT comparing IPVR with PVR. We will recruit patients being referred for pulmonary valve replacement at University Hospitals Bristol NHS Foundation Trust and University Hospitals Southampton NHS Foundation Trust.

Eligibility criteria

Potential participants will be eligible if they are:

1. Aged between 12 and 80 years.
2. Have a valve size between 25-31mm.
3. Undergoing either:
    a. Replacement of the pulmonary valve;
    b. Replacement of the pulmonary valve with atrial septal defect (ASD) which is amenable to closure via cardiac catheter or;
    c. Replacement of the pulmonary valve with right ventricular outflow tract (RVOT) reconstruction that does not require CPB if using an injectable valve.

Potential patients will be ineligible if they are:

1. Prisoners or adults lacking capacity to consent.
2. Aged between 12 and 15 years and under the care of social services.
3. Undergoing an additional procedure that requires the use of CPB (e.g. pulmonary valve or artery stenosis requiring patch reconstruction, intra-cardiac shunt, RVOT reconstruction requiring CPB, other anatomical heart corrections).
4. Diagnosed with active endocarditis.
5. Unwilling to undergo surgery involving a porcine product.
6. Unable to assent/consent.

For peer review only

Patient approach and consent

We will identify potential participants from clinic lists (elective patients) and theatre schedules (urgent patients) and they will be given an invitation letter and patient information leaflet (PIL) suitable to their age group. Parent(s)/guardian(s) of potential participants 12-15 years old will be given an invitation letter and parent/guardian information leaflet (P/GIL). Most patients and parent(s)/guardian(s) will have at least 24 hours to consider whether they would like to participate, although in some cases this time interval may be shorter, for example, patients admitted from other hospitals without prior notification to the research team. Written informed consent/assent will be obtained from the patient and/or their parent/guardian (where appropriate) prior to inclusion in the trial. Figure 1 shows the patient pathway.

Trial interventions

For both operations the heart will be exposed through a standard incision for a heart operation (median sternotomy).

For IPVR operations the main pulmonary artery and front of the heart will be dissected out. A purse-string stitch will be placed in the front of the heart and the size of valve required will be confirmed using an echocardiogram. If the main pulmonary artery is too big, it will be made smaller using a stitch to narrow it. The valve injector will be inserted into the heart through the purse string and the valve will be deployed, then sewn into position using 3 fixation stitches. If the patient becomes unstable during the surgery, CPB will be instituted. The position and function of the valve will be confirmed using transoesophageal echocardiography. The operation will then be completed in a standard manner.

For PVR operations the heart will be freed from the scar tissue and the patient put on CPB. The pulmonary artery will be opened and the old valve removed. A new valve that is of the correct size will be sewn into place; this may mean that in some cases the heart needs to be stopped for a short period of time. A patch may also be necessary to close the heart over the new valve. Once this has been completed, CPB will be discontinued when the heart has recovered from the effects of cardioplegia arrest and the operation will be completed in a standard manner.

All other aspects of participant's care will be performed according to standard practice. We will follow up participants for 6 months post-surgery.

Randomisation

Trial allocations will be generated by a computer using block randomisation, with varying block sizes. Participants will be randomly assigned in a 1:1 ratio and randomisation will be stratified by centre. If a participant's operation is unexpectedly rescheduled, the trial number and randomised allocation will be retained.

Trial outcomes

The primary outcome will be post-surgery blood loss as measured by the chest drainage volume in the first 24 post-surgical hours. The secondary outcomes include:

1) Post-surgery time-to-'readiness for extubation'. The following criteria will be documented at the time of extubation (when all criteria should be met): normal temperature, cardiovascular system stable, no metabolic imbalance, blood loss decreasing and below a defined rate, able to clear respiratory secretions, patient awake, no residual muscle paralysis and adequate analgesia;
2) Length of intensive care unit (ICU) stay;
3) Inotropic/vasodilator support;
4) Chest drain volume in the first 12 post-surgical hours;
5) Blood products used in the first 24 post-surgical hours;
(6) Post-surgery time to 'fitness for discharge' (FFD), defined as when all of: normal temperature, pulse and respiration, normal oxygen saturation on air, normal bowel function, physically mobile are met;
7) CMR assessment of valve and heart function at 6 months, defined as: presence and degree of pulmonary regurgitation, end-diastolic volume and right ventricular ejection fraction;
8) Echocardiography assessment of valve function at routine follow-up at 6 months, defined as presence and degree of pulmonary regurgitation and residual valve stenosis, measured as peak velocity through the valve;

For peer review only

9) HRQoL at 6 weeks and 6 months post-surgery (EuroQol EQ-5D in patients ≥18 years and EQ-5D-Y in patients <18 years; SF36 in patients ≥18 years and child health questionnaire (CHQ) in patients <18 years);

10) Valve-related complications during follow-up.

Data collection

The schedule of data collection is shown in Table 1. Data will be collected from medical notes and hospital records, entered onto a bespoke database and stored on a secure server.

For peer review only

Table 1. Schedule of data collection

Data item	*Pre-surgery*	*During surgery*	*Post-surgery (up to and including discharge)*	*6 weeks post-surgery*	*6 months post-surgery*
Screening and consent data	✓				
Baseline data (patient demographics, medical history, medications)	✓				
CMR imaging	✓				✓
Echocardiography	✓				✓
Randomisation allocation	✓				
Intervention (IPVR/PVR)		✓			
Inotropic/vasodilator support			✓		
Chest drain volume in the first 12 and 24 hours			✓		
Blood products used in the first 24 hours			✓		

Readiness for extubation	✓		
Intensive care unit stay	✓		
Fitness for discharge	✓		
Health related quality of life		✓	✓
Follow up questionnaires to collect post-surgery complications and safety data		✓	✓

Blinding

We will blind participants to their treatment allocation and assess the efficacy of blinding at 6 weeks and 6 months post-surgery via questionnaires. We will record any instances of unblinding. Staff responsible for the care of the patient will be aware that the patient is blinded and asked not to disclose the patient's treatment allocation. It will not be possible to blind surgeons, staff responsible for the care of the patient or research nurses to the participant's treatment allocation. Details of the procedure are needed to order an injectable valve of the required size and also needs to be recorded in the medical notes.

Sample size calculation

A sample size of 60 (30 per group) will have 80% power to detect an approximate 50% reduction in chest drainage volume (effect size on logarithmic scale of -0.73) over the first 24 hours at a 5% significance level (2 tailed). This target difference is large but is entirely consistent with the >70% reduction observed in a small non-randomised comparison comparing patients who had IPVR and those who had PVR.(11)

Statistical analysis

The trial will be analysed on an intention-to-treat basis, i.e. outcomes will be analysed according to the treatment allocation. Any non-adherence to the allocated group will be documented. The primary outcome (chest drainage volume in the first 24 post-surgical hours) will be analysed using linear regression (on the logarithmically transformed values, as the distribution is expected to be skewed). All secondary outcomes will yield either binary, quantitative, time to event or longitudinal

For peer review only

data, and will be analysed using logistic regression (binary outcomes), linear regression (quantitative outcomes), survival methods (time to event outcomes). Outcomes with repeated measures (longitudinal data) will be analysed using mixed models, which allow for unbalanced data. Alternative correlation structures will be considered, and the sensitivity of the results to the choice of structure examined. Analyses will be adjusted for centre (stratification factor). Outcomes will be reported as effect sizes with 95% confidence intervals.

Patient and Public Involvement

Patients were not actively involved in the design of the study. However, the PIL for patients aged 12-15 year olds was reviewed by 3 children and their families (the children were members of the public who did not have congenital heart disease) to confirm the acceptability of the study and that the content and format of the PIL was appropriate for this age group. Changes to the PIL were made based on their feedback to ensure the PIL was suitable for this age group.

Patients will be informed of the results through patient organisations and a summary report for participants. The results summary for participants will be designed with input from the Cardiovascular Biomedical Research Centre (CV-BRC) PPI advisory group.

Risk of bias

We have designed the trial to minimise the risk of bias. Participants will be randomised after eligibility is confirmed. Participants will be blinded to treatment allocation to ensure that patient-reported outcomes (HRQoL) are not influenced by any perceptions about which valve is best.

Although surgeons, staff responsible for the care of the patient and research nurses will not be blinded to the treatment allocation, all surgical procedures will follow standard protocols. We will pre-define all procedures and data collection required for participant follow-up and apply the procedures to all participants in the same way.

We expect minimal missing outcome data as most outcome data will be collected during the participants' hospital stay. We will maintain contact with participants during follow up (e.g. contacting patients with overdue questionnaires) to maximise

For peer review only

questionnaire completion rates and attendance at the 6 month follow up appointment.

We have pre-specified all outcomes and will write a detailed analysis plan before the database is closed and comparisons between groups are investigated to prevent selective reporting of results.

ETHICS

The trial received research ethics approval from the South West - Exeter Research Ethics Committee (REC) in November 2015. The trial is managed by the Clinical Trials and Evaluation Unit Bristol (CTEU Bristol) and sponsored by University Hospitals Bristol NHS Foundation Trust (www.uhbristol.nhs.uk/research-innovation/). Participants and their parents/guardians (where applicable) have the right to withdraw at any time and if they do withdraw, will be treated according to their hospitals' standard procedures. Participants who choose to withdraw from the study will be asked if we can retain and continue to use any data already collected and whether they are willing to participate in the trial follow up.

Changes to the protocol since REC approval

We made three major changes to the protocol before recruitment began. First, we decided to blind participants to ensure validity of the patient-reported outcome data. Secondly, we decided that it was not feasible to blind the research nurses to the treatment allocation as they would be required to order the valves. Thirdly, initially we intended to establish a Data Monitoring and Safety Committee (DMSC) to oversee the trial. Given that the trial has a relatively low target sample size, a short follow up period and we expect very few serious adverse events, it is hard to conceive of an interim analysis (e.g. after 50% of the participants have been recruited) with sufficient power to identify a safety issue that would require action to be taken. We concluded therefore that it was sufficient for the safety data to be reviewed by the Trial Management Group and Trial Steering Committee for Cardiovascular Studies, a sub-group of the University Hospitals Bristol NHS Foundation Trust Cardiovascular Research Board. Shortly after opening to recruitment, we made one major change to the protocol, to extend the baseline data

For peer review only

collection to include the pre-surgery echocardiography and CMR results. Version 5.0 (dated 13/09/2017) of the protocol is currently in use.

RECRUITMENT AND ISSUES EXPERIENCED

The InVITe trial opened to recruitment in April 2016 in Bristol and the first patient was recruited in July 2016. Recruitment in Southampton opened in January 2017. To date, we have identified 38 eligible patients, consented 17 patients, 13 of which have had surgery (8 of these have completed follow up).

Recruitment has been slower than anticipated for a number of reasons. Several sites were approached to participate in the study but have declined. Reasons given included a reluctance to depart from the traditional care pathway for high risk patients, the excess treatment costs involved, and the motivation of surgeons to undertake the study. In the recruiting sites, the number of patients being referred for a pulmonary valve replacement is lower than expected; and the number of patients who do not meet the eligibility criteria is higher than originally anticipated.

Additionally, organising training in IPVR surgery has proved logistically challenging. As part of the trial the surgeons were required to complete two training cases, which were supervised by a leading expert in IPVR surgery. It took longer than expected for all participating surgeons to complete the required training. During this time, patients whose operations were scheduled at short notice could not be approached. In Bristol, there were initially two participating surgeons. To help improve recruitment rates, we invited an additional surgeon to participate in the study.

For peer review only

DISSEMINATION OF FINDINGS

We will present our findings at international meetings and in peer-reviewed publications. We will inform the public through patient organisations and newsletters to participants.

DISCUSSION

The InVITe trial will be the first RCT to compare IPVR with PVR in patients requiring replacement of the pulmonary valve. To our knowledge, the trial remains the only RCT (published or registered) comparing IPVR with conventional PVR. The

proposed trial should therefore contribute significantly to the understanding of injectable pulmonary valve replacement and inform larger RCTs in the future.

Most individuals with congenital heart disease need repeated operations to replace the pulmonary valve. For example, if a child needs a pulmonary valve replaced, it is more likely that the prosthetic valve will need to be replaced more than once as the tissue valve will wear out. It is therefore essential to look at ways to minimise harm and improve quality of life after surgery. The main benefits for patients for IPVR are believed to be a quicker recovery as well as a lower risk of post-surgery morbidity, including serious but rare (<5%) complications. These benefits to patients are expected to translate into benefits for the NHS with fewer resources needed during and after the operation (shorter surgery time and no CPB costs, shorter duration of ventilation, intensive care and overall hospital stay; fewer blood products; fewer resources required to treat comorbidity).

The main strengths of our trial are the inclusion of both children and adults, which will ensure that our findings are generalizable to a wide range of patients, the collection of data on a wide range of primary and secondary outcomes, which could inform the selection of outcomes in a future larger RCT, and the inclusion of a patient-reported outcome (HRQoL), which is important in a population that needs repeated operations.

AUTHORS' CONTRIBUTIONS

RH: Preparation and drafting of trial protocol, writing manuscript.

GA: Trial design, review of trial protocol, review of manuscript.

MC: Participating surgeon in the trial, review of manuscript.

LC: Trial design, preparation and drafting of trial protocol, review of manuscript.

MP: Preparation and drafting of trial protocol, review of manuscript.

BR: Trial design, preparation and drafting of trial protocol, review of manuscript.

CR: Trial design, preparation and drafting of trial protocol, sample size and statistical analysis plan, review of manuscript.

SS: Participating surgeon in the trial, review of manuscript.

For peer review only

AP: Trial concept, study design, preparation of trial protocol, definition of trial interventions, definition of outcomes, writing manuscript. Chief Investigator.

All authors read and approved the final manuscript.

ACKNOWLEDGEMENTS

The InVITe trial is sponsored by University Hospitals Bristol NHS Foundation Trust.

This trial was designed and delivered in collaboration with the Clinical Trials and Evaluation Unit (CTEU), a UKCRC registered clinical trials unit which, as part of the Bristol Trials Centre is in receipt of National Institute for Health Research CTU support funding. The research team acknowledges the support of the National Institute for Health Research Clinical Research Network (NIHR CRN).

The authors would like to thank all trial team members involved in the recruitment, coordination and data entry for this trial and members of the CV-BRC PPI group and public who assisted with the review of study documents. Special thanks are also given to perfusion, anaesthetics and nursing teams for their support in the conduct of the trial and to Pierson Ltd and Stefano Marianeschi for their help in providing IPVR training

COMPETING INTERESTS

Pierson Ltd have agreed to provide the injectable tissue valves (BioPulmonic™, Biointegral Surgical Inc.) used in the trial at a discounted rate. Pierson Ltd paid for the expenses associated with Stefano Marianeschi to visit the trial centres and provide IPVR training. Pierson Ltd had no input into the design or conduct of the trial, and will have no access to the data arising from the trial.

For peer review only

FUNDING STATEMENT

The trial is funded by the National Institute for Health Research (NIHR) Bristol Cardiovascular Biomedical Research Centre (Cardiovascular theme) and the British Heart Foundation. The views and opinions expressed herein are those of the authors and do not necessarily reflect those of the NIHR, NHS or the Department of Health.

Additional Figures

Figure 1. Trial Schema showing the recruitment pathway with the number of patients to be recruited, anticipated eligibility and recruitment rates.

LIST OF ABBREVIATIONS

ASD	Atrial septal defect
CMR	Cardiovascular magnetic resonance imaging
CPB	Cardiopulmonary bypass - a technique that temporarily takes over the function of the heart and lungs during surgery, maintaining the circulation of blood and the oxygen content of the body whilst allowing surgeons to access static cardiac tissue.
CHQ	Child Health Questionnaire
CTEU	Clinical Trials and Evaluation Unit
DMSC	Data monitoring and safety committee
EQ-5D	EuroQoL-5D
EQ-5D-Y	EuroQoL-5D - (Youth)
FFD	Fitness for discharge
HRQoL	Health related quality of life
ICU	Intensive care unit
IPVR	Injectable pulmonary valve replacement
NIHR	National Institute for Health Research
PIL	Patient information leaflet
P/GIL	Parent/Guardian information leaflet
PVR	Pulmonary valve replacement (conventional)

For peer review only

RCT	Randomised controlled trial
REC	Research ethics committee
RVOT	Right ventricle outflow tract
SF36	36-Item Short Form Survey

REFERENCES

1. Sanchez HE, Cornish EM, Shih FC, et al. The surgical treatment of tetralogy of Fallot. *Ann Thorac Surg.* 1984;37(5):431-6.
2. National Congenital Heart Disease Audit Website 2016 [Available from: https://nicor4.nicor.org.uk/chd/an_paeds.nsf/0/E77F61C7064DA941802580EC0027E335.
3. Berdat PA, Carrel T. Off-pump pulmonary valve replacement with the new Shelhigh Injectable Stented Pulmonic Valve. J Thorac Cardiovasc Surg. 2006;131(5):1192-3.
4. Dittrich S, Gloeckler M, Arnold R, et al. Hybrid pulmonary valve implantation: injection of a self-expanding tissue valve through the main pulmonary artery. *Ann Thorac Surg.* 2008;85(2):632-4.
5. Schreiber C, Horer J, Vogt M, et al. A new treatment option for pulmonary valvar insufficiency: first experiences with implantation of a self-expanding stented valve without use of cardiopulmonary bypass. *Eur J Cardiothorac Surg.* 2007;31(1):26-30.
6. Boone RH, Webb JG, Horlick E, et al. Transcatheter pulmonary valve implantation using the Edwards SAPIEN transcatheter heart valve. *Catheter Cardiovasc Interv.* 2010;75(2):286-94.
7. von Wattenwyl R, Arnold R, Siepe M, Implantation of a catheter-based self-expanding pulmonary valve in congenital heart surgery: results of a pilot study. *Eur J Cardiothorac Surg.* 2011;40(3):552-6.
8. Marianeschi SM, Santoro F, Ribera E, et al. Pulmonary valve implantation with the new Shelhigh Injectable Stented Pulmonic Valve. *Ann Thorac Surg.* 2008;86(5):1466-71; discussion 72.
9. Deorsola L, Abbruzzese PA. Use of oversized injectable valves in growing children for total repair of right ventricular outflow tract anomalies (preliminary results). Tex Heart Inst J. 2014;41(4):373-80.
10. Grohmann J, Hohn R, Fleck T, et al. No-React(R) Injectable BioPulmonic valves re-evaluated: discouraging follow-up results. *Interact Cardiovasc Thorac Surg.* 2015;21(5):657-65.
11. Chen Q, Turner M, Caputo M, et al. Pulmonary valve implantation using self-expanding tissue valve without cardiopulmonary bypass reduces operation time and blood product use. *J Thorac Cardiovasc Surg*. 2013;145(4):1040-5.

For peer review only

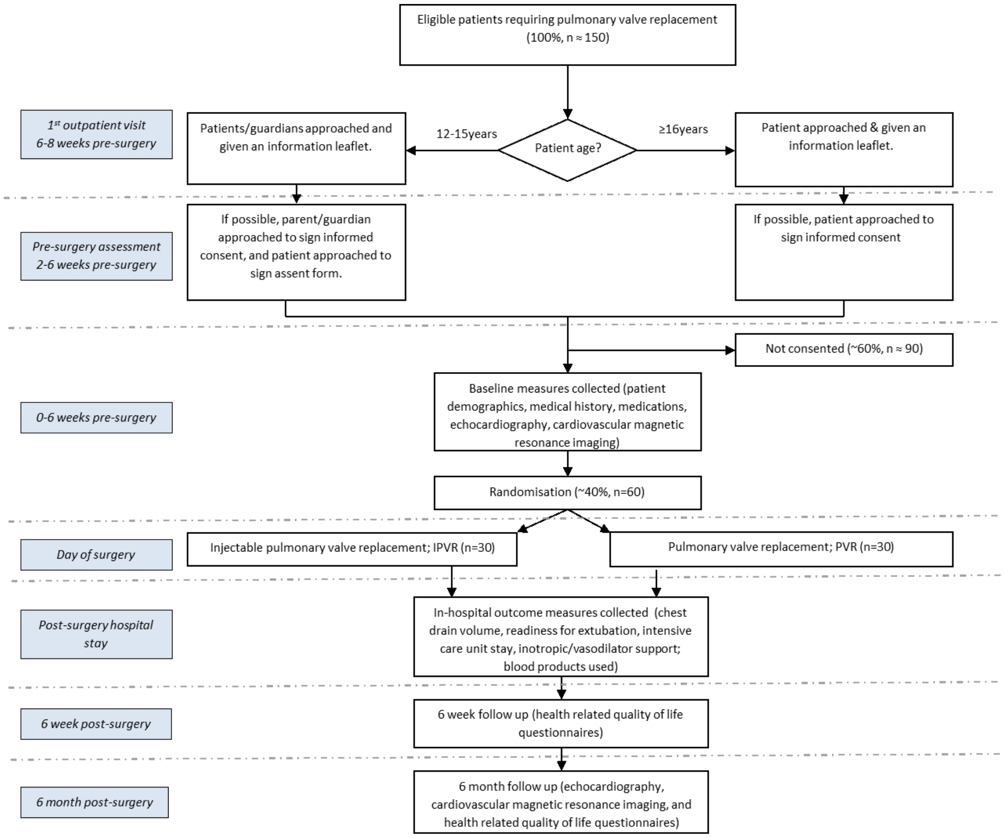

Figure 1. Trial Schema showing the recruitment pathway with the number of patients to be recruited, anticipated eligibility and recruitment rates.

108x90mm (300 x 300 DPI)

SPIRIT

STANDARD PROTOCOL ITEMS: RECOMMENDATIONS FOR INTERVENTIONAL TRIALS

SPIRIT 2013 Checklist: Recommended items to address in a clinical trial protocol and related documents*

Section/item	Item No	Description	Addressed on page number
**Administrative information**			
Title	1	Descriptive title identifying the study design, population, interventions, and, if applicable, trial acronym	_______1_______
Trial registration	2a	Trial identifier and registry name. If not yet registered, name of intended registry	_______3_______
	2b	All items from the World Health Organization Trial Registration Data Set	_____n/a for protocol paper
Protocol version	3	Date and version identifier	_______12_______
Funding	4	Sources and types of financial, material, and other support	_______15_______
Roles and responsibilities	5a	Names, affiliations, and roles of protocol contributors	_____1,14____
	5b	Name and contact information for the trial sponsor	_____15_______
	5c	Role of study sponsor and funders, if any, in study design; collection, management, analysis, and interpretation of data; writing of the report; and the decision to submit the report for publication, including whether they will have ultimate authority over any of these activities	_____n/a______
	5d	Composition, roles, and responsibilities of the coordinating centre, steering committee, endpoint adjudication committee, data management team, and other individuals or groups overseeing the trial, if applicable (see Item 21a for data monitoring committee)	_______n/a_____

For peer review only

Introduction

Background and rationale	6a	Description of research question and justification for undertaking the trial, including summary of relevant studies (published and unpublished) examining benefits and harms for each intervention	____4,5_______
	6b	Explanation for choice of comparators	______5______
Objectives	7	Specific objectives or hypotheses	_____5_______
Trial design	8	Description of trial design including type of trial (eg, parallel group, crossover, factorial, single group), allocation ratio, and framework (eg, superiority, equivalence, noninferiority, exploratory)	______6,7_____

Methods: Participants, interventions, and outcomes

Study setting	9	Description of study settings (eg, community clinic, academic hospital) and list of countries where data will be collected. Reference to where list of study sites can be obtained	______5______
Eligibility criteria	10	Inclusion and exclusion criteria for participants. If applicable, eligibility criteria for study centres and individuals who will perform the interventions (eg, surgeons, psychotherapists)	______5_______
Interventions	11a	Interventions for each group with sufficient detail to allow replication, including how and when they will be administered	______7,8______
	11b	Criteria for discontinuing or modifying allocated interventions for a given trial participant (eg, drug dose change in response to harms, participant request, or improving/worsening disease)	_____12_______
	11c	Strategies to improve adherence to intervention protocols, and any procedures for monitoring adherence (eg, drug tablet return, laboratory tests)	________n/a_____
	11d	Relevant concomitant care and interventions that are permitted or prohibited during the trial	______6_______
Outcomes	12	Primary, secondary, and other outcomes, including the specific measurement variable (eg, systolic blood pressure), analysis metric (eg, change from baseline, final value, time to event), method of aggregation (eg, median, proportion), and time point for each outcome. Explanation of the clinical relevance of chosen efficacy and harm outcomes is strongly recommended	______8-10_____
Participant timeline	13	Time schedule of enrolment, interventions (including any run-ins and washouts), assessments, and visits for participants. A schematic diagram is highly recommended (see Figure)	_____9,10______

For peer review only - http://bmjopen.bmj.com/site/about/guidelines.xhtml

Sample size	14	Estimated number of participants needed to achieve study objectives and how it was determined, including clinical and statistical assumptions supporting any sample size calculations	______10_______
Recruitment	15	Strategies for achieving adequate participant enrolment to reach target sample size	_____12,13_____

Methods: Assignment of interventions (for controlled trials)

Allocation:

Sequence generation	16a	Method of generating the allocation sequence (eg, computer-generated random numbers), and list of any factors for stratification. To reduce predictability of a random sequence, details of any planned restriction (eg, blocking) should be provided in a separate document that is unavailable to those who enrol participants or assign interventions	_____7_______
Allocation concealment mechanism	16b	Mechanism of implementing the allocation sequence (eg, central telephone; sequentially numbered, opaque, sealed envelopes), describing any steps to conceal the sequence until interventions are assigned	_______7______
Implementation	16c	Who will generate the allocation sequence, who will enrol participants, and who will assign participants to interventions	_______7______
Blinding (masking)	17a	Who will be blinded after assignment to interventions (eg, trial participants, care providers, outcome assessors, data aalysts), and how	_____10________
	17b	If blinded, circumstances under which unblinding is permissible, and procedure for revealing a participant's allocated intervention during the trial	NI

Methods: Data collection, management, and analysis

Data collection methods	18a	Plans for assessment and collection of outcome, baseline, and other trial data, including any related processes to promote data quality (eg, duplicate measurements, training of assessors) and a description of study instruments (eg, questionnaires, laboratory tests) along with their reliability and validity, if known. Reference to where data collection forms can be found, if not in the protocol	_____9,10______
	18b	Plans to promote participant retention and complete follow-up, including list of any outcome data to be collected for participants who discontinue or deviate from intervention protocols	11

Data management	19	Plans for data entry, coding, security, and storage, including any related processes to promote data quality (eg, double data entry; range checks for data values). Reference to where details of data management procedures can be found, if not in the protocol	_____N/I______
Statistical methods	20a	Statistical methods for analysing primary and secondary outcomes. Reference to where other details of the statistical analysis plan can be found, if not in the protocol	_____10,11___
	20b	Methods for any additional analyses (eg, subgroup and adjusted analyses)	_____N/A______
	20c	Definition of analysis population relating to protocol non-adherence (eg, as randomised analysis), and any statistical methods to handle missing data (eg, multiple imputation)	_____N/I_____
**Methods: Monitoring**			
Data monitoring	21a	Composition of data monitoring committee (DMC); summary of its role and reporting structure; statement of whether it is independent from the sponsor and competing interests; and reference to where further details about its charter can be found, if not in the protocol. Alternatively, an explanation of why a DMC is not needed	______12_______
	21b	Description of any interim analyses and stopping guidelines, including who will have access to these interim results and make the final decision to terminate the trial	______N/A_____
Harms	22	Plans for collecting, assessing, reporting, and managing solicited and spontaneously reported adverse events and other unintended effects of trial interventions or trial conduct	______N/I_______
Auditing	23	Frequency and procedures for auditing trial conduct, if any, and whether the process will be independent from investigators and the sponsor	______N/I_______
**Ethics and dissemination**			
Research ethics approval	24	Plans for seeking research ethics committee/institutional review board (REC/IRB) approval	_____11______
Protocol amendments	25	Plans for communicating important protocol modifications (eg, changes to eligibility criteria, outcomes, analyses) to relevant parties (eg, investigators, REC/IRBs, trial participants, trial registries, journals, regulators)	_____N/I________

For peer review only - http://bmjopen.bmj.com/site/about/guidelines.xhtml

Consent or assent	26a	Who will obtain informed consent or assent from potential trial participants or authorised surrogates, and how (see Item 32)	_______7_____
	26b	Additional consent provisions for collection and use of participant data and biological specimens in ancillary studies, if applicable	_____N/A________
Confidentiality	27	How personal information about potential and enrolled participants will be collected, shared, and maintained in order to protect confidentiality before, during, and after the trial	______N/I________
Declaration of interests	28	Financial and other competing interests for principal investigators for the overall trial and each study site	______15_______
Access to data	29	Statement of who will have access to the final trial dataset, and disclosure of contractual agreements that limit such access for investigators	_______N/I_____
Ancillary and post-trial care	30	Provisions, if any, for ancillary and post-trial care, and for compensation to those who suffer harm from trial participation	_______N/A_______ _
Dissemination policy	31a	Plans for investigators and sponsor to communicate trial results to participants, healthcare professionals, the public, and other relevant groups (eg, via publication, reporting in results databases, or other data sharing arrangements), including any publication restrictions	______3________
	31b	Authorship eligibility guidelines and any intended use of professional writers	_______N/A_______
	31c	Plans, if any, for granting public access to the full protocol, participant-level dataset, and statistical code	______N/A________
**Appendices**			
Informed consent materials	32	Model consent form and other related documentation given to participants and authorised surrogates	On request
Biological specimens	33	Plans for collection, laboratory evaluation, and storage of biological specimens for genetic or molecular analysis in the current trial and for future use in ancillary studies, if applicable	______N/A_______

*It is strongly recommended that this checklist be read in conjunction with the SPIRIT 2013 Explanation & Elaboration for important clarification on the items. Amendments to the protocol should be tracked and dated. The SPIRIT checklist is copyrighted by the SPIRIT Group under the Creative Commons "Attribution-NonCommercial-NoDerivs 3.0 Unported" license.

'Off pump' self-expanding injectable tissue valves (IPVR) versus 'on pump' conventional tissue valves (PVR) for replacement of the pulmonary valve: trial protocol for a randomised controlled trial (InVITe trial)

Journal:	*BMJ Open*
Manuscript ID	bmjopen-2018-026221.R1
Article Type:	Protocol
Date Submitted by the Author:	27-Nov-2018
Complete List of Authors:	Heys, Rachael; University of Bristol , Clinical Trials and Evaluation Unit, Bristol Trials Centre, Bristol Medical School<br>Angelini, Gianni; University of Bristol, Bristol Heart Institute<br>Caputo, Massimo; University Hospitals Bristol NHS Foundation Trust, Bristol Royal Hospital for Children<br>Culliford, Lucy; University of Bristol , Clinical Trials and Evaluation Unit, Bristol Trials Centre, Bristol Medical School<br>Pufulete, Maria; University of Bristol, Clinical Trials and Evaluation Unit, Bristol Trials Centre, Bristol Medical School<br>Reeves, Barnaby; University of Bristol, Clinical Trials and Evaluation Unit, Bristol Trials Centre, Bristol Medical School<br>Rogers, Chris; University of Bristol , Clinical Trials and Evaluation Unit, Bristol Trials Centre, Bristol Medical School<br>Stoica, Serban ; University Hospitals Bristol NHS Foundation Trust<br>Parry , Andrew ; University Hospitals Bristol NHS Foundation Trust, Bristol Royal Hospital for Children
**Primary Subject Heading**:	Cardiovascular medicine
Secondary Subject Heading:	Surgery
Keywords:	Cardiac surgery < SURGERY, Congenital heart disease < CARDIOLOGY, Valvular heart disease < CARDIOLOGY, Protocols & guidelines < HEALTH SERVICES ADMINISTRATION & MANAGEMENT

SCHOLARONE™
Manuscripts

For peer review only - http://bmjopen.bmj.com/site/about/guidelines.xhtml

‘Off pump’ self-expanding injectable tissue valves (IPVR) versus ‘on pump’ conventional tissue valves (PVR) for replacement of the pulmonary valve: trial protocol for a randomised controlled trial (InVITe trial)

Rachael Heys[1] rachael.heys@bristol.ac.uk

Gianni Angelini[2] g.d.angelini@bristol.ac.uk

Massimo Caputo[3] m.caputo@bristol.ac.uk

Lucy Culliford[1] lucy.culliford@bristol.ac.uk

Maria Pufulete[1] maria.pufulete@bristol.ac.uk

Barnaby C Reeves[1] barney.reeves@bristol.ac.uk

Chris A Rogers[1] chris.rogers@bristol.ac.uk

Serban Stoica[3] serban.stoica@uhbristol.nhs.uk

Andrew Parry[2] andrew.parry@uhbristol.nhs.uk

1. Clinical Trials and Evaluation Unit, Bristol Trials Centre, Bristol Medical School, University of Bristol, Bristol, UK
2. Bristol Heart Institute, University of Bristol, Bristol, UK
3. Bristol Royal Hospital for Children, University Hospitals Bristol, Bristol, UK

Corresponding author:

Mr Andrew Parry, Bristol Royal Hospital for Children, University Hospitals Bristol, Bristol, UK. Email: Andrew.parry@uhbristol.nhs.uk. Tel: 0117 342 8854

Word count: 3208 (excluding title page, abstract, references, figures and tables)

Number of figures: 1

Number of tables: 1

For peer review only

Keywords: Pulmonary valve; congenital; cardiopulmonary bypass (CPB); self-expanding injectable valve; minimally invasive; cardiac surgery; no-react injectable BioPulmonic valve; randomised controlled trial.

For peer review only

ABSTRACT

Introduction: Patients with congenital heart disease often need repeated operations throughout life to replace the pulmonary valve. Valve replacement with 'injectable' self-expanding valves (which is performed 'off pump'; without the use of cardiopulmonary bypass, CPB) may result in quicker recovery and lower risk of major complications than valve replacement with conventional valves (which is performed 'on pump', with the use of CPB).

Methods and analysis: We are conducting a multi-centre, single-blind randomised controlled trial in patients aged between 12 and 80 years with congenital heart disease. We will randomise participants in a 1:1 ratio to receive either 'off pump' injectable pulmonary valve replacement or 'on pump' conventional pulmonary valve replacement. The primary outcome will be the difference between groups with respect to post-surgery blood loss (as measured by chest drain volume) in the first 24 hours. Secondary outcomes will include in-hospital outcomes (intensive care unit stay; inotropic/vasodilator support; chest drain volume in the first 12 hours post-surgery; time of readiness for extubation; blood products used in the first 24 hours post-surgery; time of fitness for discharge); valve and heart function 6 months post-surgery (assessed using cardiovascular magnetic resonance and echocardiography); and health related quality of life 6 weeks and 6 months post-surgery.

Ethics and dissemination: This trial has been approved by the South West – Exeter Research Ethics Committee. Findings will be shared with participating hospitals and disseminated to the academic community through peer reviewed publications and presentation at national and international meetings. Patients will be informed of the results through patient organisations and newsletters to participants.

Trial registration: ISRCTN registry, identifier ISRCTN23538073. Registered on 20/01/2016.

Strengths and limitations

- This trial is the first randomised controlled trial (RCT) to assess the effectiveness of injectable tissue valves versus conventional tissue valves for replacement of the pulmonary valve.
- This is a multi-centre RCT, with computer-generated concealed randomisation and participant blinding to minimise bias.
- We are including both children and adults (age range 12-80 years) to ensure the findings will be generalizable to a wide group of patients who need pulmonary valve replacement.
- Although clinicians cannot be blinded to the patient's treatment allocation we will minimise bias by pre-defining all procedures for data collection and applying these to all participants in the same way.
- The primary and many of the secondary endpoints are clinical outcomes, based on objective criteria, which are important to patients, clinical teams, and the NHS.

INTRODUCTION

Background and objectives

Most children born with complex congenital heart disease now reach adulthood. Post-surgery mortality following surgical correction of Tetralogy of Fallot for example, has fallen from 5.5% in 1980, (1) to 0% in 2016.(2) However, as long term survival has improved late sequelae have become evident. Many of the patients' operated on for tetralogy of Fallot and other forms of pulmonary valve disease require multiple operations throughout life to repair or replace poorly functioning pulmonary valves.

The standard operation for pulmonary valve replacement (PVR) involves opening the chest, exposing the heart and using cardiopulmonary bypass (CPB; 'on pump'). CPB is a technique that temporarily takes over the function of the heart and lungs (pumping blood and oxygen through the body) and occasionally involves stopping the heart during surgery. Use of CPB is associated with complications such as excessive bleeding and reperfusion injury in cases where the heart has to be stopped (damage to heart tissue occurs when the blood supply returns to the heart).

For peer review only

Injectable tissue valves (BioPulmonic™, Biointegral Surgical Inc.) have been developed to replace conventional tissue valves for PVR. Injectable tissue valves can be implanted without the use of CPB ('off pump') and with less dissection of the heart. Case reports and small case series (<12 patients)(3-10) have shown that injectable tissue valves are easy to implant and have satisfactory function. Pilot data have shown shorter operating time (2 hours less), and less blood loss (over 400 ml less) and blood product requirement (3 units less) in 6 'off-pump' injectable pulmonary valve replacement (IPVR) patients compared with 7 'on pump' PVR patients. No patient had a paravalvular leak (leak around the replaced valve) or more than mild pulmonary regurgitation at early follow up, suggesting that IPVR is a safe procedure.(11) There are no randomised controlled trials (RCTs) that have evaluated IPVR versus PVR.

We are conducting an RCT to determine whether IPVR leads to less blood loss, faster recovery, lower risk of post-surgery complications and good valve function in patients who need an operation to replace their pulmonary valve. We could not consider percutaneous transcatheter pulmonary valve replacement (TPVR), which is performed via cardiac catheterisation and therefore does not require open-heart surgery, because current TVPR valve sizes are only suitable for patients left ventricular outflow tract (RVOT) diameter of <=27mm, which would exclude >80% of our eligible population.(12, 13) Further, all the eligible patients will have native outflow tracts that are too distensile for robust fixation of a percutaneous valve.

AIMS AND OBJECTIVES

The main aim of the InVITe trial is to compare IPVR versus PVR in patients that need pulmonary valve replacement. The objectives are to estimate the difference between groups with respect to:

1. Post-surgery blood loss (as measured by chest drain volume) in the first 24 hours (primary outcome);
2. A range of clinical post-surgery outcomes (intensive care unit (ICU) stay; inotropic/vasodilator support; chest drain volume in the first 12 hours; time

For peer review only

until ready for extubation; blood products used in the first 24 hours; fitness for discharge (FFD); valve-related complications during follow-up);

3. Heart and valve function assessed using cardiovascular magnetic resonance (CMR) and echocardiography;
4. Health related quality of life (HRQoL).

METHODS

Trial design and population

The InVITe trial is an early phase, multi-centre, RCT comparing IPVR with PVR. We will recruit patients being referred for pulmonary valve replacement at University Hospitals Bristol NHS Foundation Trust and University Hospitals Southampton NHS Foundation Trust.

Eligibility criteria

Potential participants will be eligible if they are:

1. Aged between 12 and 80 years.
2. Have a valve size between 25-31mm.
3. Undergoing any of the following as either a first or redo procedure:
    a. Replacement of the pulmonary valve;
    b. Replacement of the pulmonary valve with atrial septal defect (ASD) which is amenable to closure via cardiac catheter or;
    c. Replacement of the pulmonary valve with right ventricular outflow tract (RVOT) reconstruction that does not require CPB if using an injectable valve.

Potential patients will be ineligible if they are:

1. Prisoners or adults lacking capacity to consent.
2. Aged between 12 and 15 years and under the care of social services.
3. Undergoing an additional procedure that requires the use of CPB (e.g. pulmonary valve or artery stenosis requiring patch reconstruction, intra-cardiac shunt, RVOT reconstruction requiring CPB, other anatomical heart corrections).

For peer review only

4. Diagnosed with active endocarditis.
5. Unwilling to undergo surgery involving a porcine product.
6. Unable to assent/consent.

Patient approach and consent

We will identify potential participants from clinic lists (elective patients) and theatre schedules (urgent patients) and they will be given an invitation letter and patient information leaflet (PIL) suitable to their age group. Parent(s)/guardian(s) of potential participants 12-15 years old will be given an invitation letter and parent/guardian information leaflet (P/GIL). Most patients and parent(s)/guardian(s) will have at least 24 hours to consider whether they would like to participate, although in some cases this time interval may be shorter, for example, patients admitted from other hospitals without prior notification to the research team. Written informed consent/assent will be obtained from the patient and/or their parent/guardian (where appropriate) prior to inclusion in the trial. Figure 1 shows the patient pathway.

Trial interventions

For both operations the heart will be exposed through a standard incision for a heart operation (median sternotomy).

For IPVR operations a Self-Expanding No-React Injectable BioPulmonic™valve (Biointegral Surgical Inc.) will be used. The main pulmonary artery and front of the heart will be dissected out. A purse-string stitch will be placed in the front of the heart and the size of valve required will be confirmed using an echocardiogram. If the main pulmonary artery is too big, it will be made smaller using a stitch to narrow it. The valve injector will be inserted into the heart through the purse string and the valve will be deployed, then sewn into position using 3 fixation stitches. If the patient becomes unstable during the surgery, CPB will be instituted. The position and function of the valve will be confirmed using transoesophageal echocardiography. The operation will then be completed in a standard manner.

For PVR operations the type of tissue valve used will be according to usual practise at the participating site and may include stented tissue valves (in adults particularly)

or homografts (in children particularly). The heart will be freed from the scar tissue and the patient put on CPB. The pulmonary artery will be opened and the old valve removed. A new valve that is of the correct size will be sewn into place; this may mean that in some cases the heart needs to be stop ped for a short period of time. A patch may also be necessary to close the heart over the new valve. Once this has been completed, CPB will be discontinued when the heart has recovered from the effects of cardioplegia arrest and the operation will be completed in a standard manner.

All other aspects of participant's care will be performed according to standard practice.

Participants will be followed up at 6 weeks (questionnaire completion) and 6 months (questionnaire completion, echocardiography and CMR) post-surgery. The 6-month echocardiography and CMR will coincide with routine hospital follow up so we expect minimal loss to follow up. A six month follow up period will provide adequate data to inform early recovery.

Randomisation

Trial allocations will be generated by a computer using block randomisation, with varying block sizes. Participants will be randomly assigned in a 1:1 ratio and randomisation will be stratified by centre. If a participant's operation is unexpectedly rescheduled, the trial number and randomised allocation will be retained.

Trial outcomes

The primary outcome will be post-surgery blood loss as measured by the chest drainage volume in the first 24 post-surgical hours. The secondary outcomes include:

1) Post-surgery time-to-'readiness for extubation'. The following criteria will be documented at the time of extubation (when all criteria should be met): normal temperature, cardiovascular system stable, no metabolic imbalance, blood loss decreasing and below a defined rate, able to clear respiratory secretions, patient awake, no residual muscle paralysis and adequate analgesia;
2) Length of intensive care unit (ICU) stay;
3) Inotropic/vasodilator support;

For peer review only

4) Chest drain volume in the first 12 post-surgical hours;

5) Blood products used in the first 24 post-surgical hours (red blood cells, fresh frozen plasma (FFP), platelets, human albumin solution (HAS), cryoprecipitate) and bleeding events that may or may not lead to re-operation;

(6) Post-surgery time to ‘fitness for discharge’ (FFD), defined as when all of: normal temperature, pulse and respiration, normal oxygen saturation on air, normal bowel function, physically mobile are met;

7) CMR assessment of valve and heart function at 6 months, defined as: presence and degree of pulmonary regurgitation, end-diastolic volume and right ventricular ejection fraction;

8) Echocardiography assessment of valve function at routine follow-up at 6 months, defined as presence and degree of pulmonary regurgitation and residual valve stenosis, measured as peak velocity through the valve;

9) HRQoL at 6 weeks and 6 months post-surgery (EuroQol EQ-5D in patients ≥18 years and EQ-5D-Y in patients <18 years; SF36 in patients ≥18 years and child health questionnaire (CHQ) in patients <18 years);

10) Valve-related complications up to 6 months post-surgery.

For peer review only

Data collection

The schedule of data collection is shown in Table 1. Data will be collected from medical notes and hospital records, entered onto a bespoke database and stored on a secure server. Access to the database will be limited to authorised personnel. Data will be collected and retained in accordance with the UK Data Protection Act 2018.

Table 1. Schedule of data collection

Data item	***Pre-surgery***	***During surgery***	***Post-surgery (up to and including discharge)***	***6 weeks post-surgery***	***6 months post-surgery***
Screening and consent data	✓				

Baseline data (patient demographics, medical history, medications)	✓				
CMR imaging	✓				✓
Echocardiography	✓				✓
Randomisation allocation	✓				
Intervention (IPVR/PVR)		✓			
Inotropic/vasodilator support			✓		
Chest drain volume in the first 12 and 24 hours			✓		
Blood products used in the first 24 hours			✓		
Readiness for extubation			✓		
Intensive care unit stay			✓		
Fitness for discharge			✓		
Health related quality of life				✓	✓
Follow up questionnaires to collect all serious post-surgery complications and safety data				✓	✓

Blinding

We will blind participants to their treatment allocation and assess the efficacy of blinding at 6 weeks and 6 months post-surgery via questionnaires. We will record any instances of unblinding. Staff responsible for the care of the patient will be aware that the patient is blinded and asked not to disclose the patient's treatment allocation. It will not be possible to blind surgeons, staff responsible for the care of the patient or research nurses to the participant's treatment allocation. Details of the procedure are needed to order an injectable valve of the required size and also needs to be recorded in the medical notes.

For peer review only

Sample size calculation

A sample size of 60 (30 per group) will have 80% power to detect an approximate 50% reduction in chest drainage volume (effect size on logarithmic scale of -0.73) over the first 24 hours at a 5% significance level (2 tailed). This target difference is large but is entirely consistent with the >70% reduction observed in a small non-randomised comparison comparing patients who had IPVR and those who had PVR.(11)

Statistical analysis

The trial will be analysed on an intention-to-treat basis, i.e. outcomes will be analysed according to the treatment allocation. Any non-adherence to the allocated group will be documented. The primary outcome (chest drainage volume in the first 24 post-surgical hours) will be analysed using linear regression (on the logarithmically transformed values, as the distribution is expected to be skewed). All secondary outcomes will yield either binary, quantitative, time to event or longitudinal data, and will be analysed using logistic regression (binary outcomes), linear regression (quantitative outcomes), survival methods (time to event outcomes). Outcomes with repeated measures (longitudinal data) will be analysed using mixed models, which allow for unbalanced data. Alternative correlation structures will be considered, and the sensitivity of the results to the choice of structure examined. Analyses will be adjusted for centre (stratification factor). Outcomes will be reported as effect sizes with 95% confidence intervals.

Patient and Public Involvement

Patients were not actively involved in the design of the study. However, the PIL for patients aged 12-15 year olds was reviewed by 3 children and their families (the children were members of the public who did not have congenital heart disease) to confirm the acceptability of the study and that the content and format of the PIL was appropriate for this age group. Changes to the PIL were made based on their feedback to ensure the PIL was suitable for this age group.

Patients will be informed of the results through patient organisations and a summary report for participants. The results summary for participants will be designed with input from the Cardiovascular Biomedical Research Centre (CV-BRC) PPI advisory group.

For peer review only

Risk of bias

We have designed the trial to minimise the risk of bias. Participants will be randomised after eligibility is confirmed. Participants will be blinded to treatment allocation to ensure that patient-reported outcomes (HRQoL) are not influenced by any perceptions about which valve is best.

Although surgeons, staff responsible for the care of the patient and research nurses will not be blinded to the treatment allocation, all surgical procedures will follow standard protocols. We will pre-define all procedures and data collection required for participant follow-up and apply the procedures to all participants in the same way.

We expect minimal missing outcome data as most outcome data will be collected during the participants' hospital stay. We will maintain contact with participants during follow up (e.g. contacting patients with overdue questionnaires) to maximise questionnaire completion rates and attendance at the 6 month follow up appointment.

We have pre-specified all outcomes and will write a detailed analysis plan before the database is closed and comparisons between groups are investigated to prevent selective reporting of results.

ETHICS

The trial received research ethics approval from the South West - Exeter Research Ethics Committee (REC) in November 2015. The trial is managed by the Clinical Trials and Evaluation Unit Bristol (CTEU Bristol) and sponsored by University Hospitals Bristol NHS Foundation Trust (www.uhbristol.nhs.uk/research-innovation/). Participants and their parents/guardians (where applicable) have the right to withdraw at any time and if they do withdraw, will be treated according to their hospitals' standard procedures. Participants who choose to withdraw from the study will be asked if we can retain and continue to use any data already collected and whether they are willing to participate in the trial follow up.

For peer review only

Changes to the protocol since REC approval

We made three major changes to the protocol before recruitment began. First, we decided to blind participants to ensure validity of the patient-reported outcome data. Secondly, we decided that it was not feasible to blind the research nurses to the treatment allocation as they would be required to order the valves. Thirdly, initially we intended to establish a Data Monitoring and Safety Committee (DMSC) to oversee the trial. Given that the trial has a relatively low target sample size, a short follow up period and we expect very few serious adverse events, it is hard to conceive of an interim analysis (e.g. after 50% of the participants have been recruited) with sufficient power to identify a safety issue that would require action to be taken. We concluded therefore that it was sufficient for the safety data to be reviewed by the Trial Management Group and Trial Steering Committee for Cardiovascular Studies, a sub-group of the University Hospitals Bristol NHS Foundation Trust Cardiovascular Research Board. Shortly after opening to recruitment, we made one major change to the protocol, to extend the baseline data collection to include the pre-surgery echocardiography and CMR results. Finally, the protocol and study documents have been amended to allow the central analysis of CMR data. Version 6.0 (dated 02/07/2018) of the protocol is currently in use. The relevant regulatory approvals will be obtained for amendments to the protocol and study information leaflets. Relevant parties (e.g. investigators at participating sites) will be informed via email. If changes are made to the study processes, participants will be re-consented if necessary.

RECRUITMENT AND ISSUES EXPERIENCED

The InVITe trial opened to recruitment in April 2016 in Bristol and the first patient was recruited in July 2016. Recruitment in Southampton opened in January 2017. To date, we have identified 38 eligible patients, consented 17 patients, 13 of which have had surgery (8 of these have completed follow up).

Recruitment has been slower than anticipated for a number of reasons. Several sites were approached to participate in the study but have declined. Reasons given included a reluctance to depart from the traditional care pathway for high risk patients, the excess treatment costs involved, and the motivation of surgeons to undertake the study. In the recruiting sites, the number of patients being referred for

For peer review only

a pulmonary valve replacement is lower than expected; and the number of patients who do not meet the eligibility criteria is higher than originally anticipated.

Additionally, organising training in IPVR surgery has proved logistically challenging. As part of the trial the surgeons were required to complete two training cases, which were supervised by a leading expert in IPVR surgery. It took longer than expected for all participating surgeons to complete the required training. During this time, patients whose operations were scheduled at short notice could not be approached. In Bristol, there were initially two participating surgeons. To help improve recruitment rates, we invited an additional surgeon to participate in the study.

DISSEMINATION OF FINDINGS

We will present our findings at international meetings and in peer-reviewed publications. We will inform the public through patient organisations and newsletters to participants.

DISCUSSION

The InVITe trial will be the first RCT to compare IPVR with PVR in patients requiring replacement of the pulmonary valve. To our knowledge, the trial remains the only RCT (published or registered) comparing IPVR with conventional PVR. The proposed trial should therefore contribute significantly to the understanding of injectable pulmonary valve replacement and inform larger RCTs in the future.

Most individuals with congenital heart disease need repeated operations to replace the pulmonary valve. For example, if a child needs a pulmonary valve replaced, it is more likely that the prosthetic valve will need to be replaced more than once as the tissue valve will wear out. It is therefore essential to look at ways to minimise harm and improve quality of life after surgery. The main benefits for patients for IPVR are believed to be a quicker recovery as well as a lower risk of post-surgery morbidity, including serious but rare (<5%) complications. These benefits to patients are expected to translate into benefits for the NHS with fewer resources needed during and after the operation (shorter surgery time and no CPB costs, shorter duration of ventilation, intensive care and overall hospital stay; fewer blood products; fewer resources required to treat comorbidity).

For peer review only

The main strengths of our trial are the inclusion of both children and adults, which will ensure that our findings are generalizable to a wide range of patients, the collection of data on a wide range of primary and secondary outcomes, which could inform the selection of outcomes in a future larger RCT, and the inclusion of a patient-reported outcome (HRQoL), which is important in a population that needs repeated operations.

AUTHORS' CONTRIBUTIONS

RH: Preparation and drafting of trial protocol, writing manuscript.

GA: Trial design, review of trial protocol, review of manuscript.

MC: Participating surgeon in the trial, review of manuscript.

LC: Trial design, preparation and drafting of trial protocol, review of manuscript.

MP: Preparation and drafting of trial protocol, review of manuscript.

BR: Trial design, preparation and drafting of trial protocol, review of manuscript.

CR: Trial design, preparation and drafting of trial protocol, sample size and statistical analysis plan, review of manuscript.

SS: Participating surgeon in the trial, review of manuscript.

AP: Trial concept, study design, preparation of trial protocol, definition of trial interventions, definition of outcomes, writing manuscript. Chief Investigator.

All authors read and approved the final manuscript.

ACKNOWLEDGEMENTS

The InVITe trial is sponsored by University Hospitals Bristol NHS Foundation Trust. The sponsor is responsible for the oversight of the InVITe study and will ensure the trial is managed appropriately.

This trial was designed and delivered in collaboration with the Clinical Trials and Evaluation Unit (CTEU), a UKCRC registered clinical trials unit which, as part of the Bristol Trials Centre is in receipt of National Institute for Health Research CTU

For peer review only

support funding. The research team acknowledges the support of the National Institute for Health Research Clinical Research Network (NIHR CRN).

The authors would like to thank all trial team members involved in the recruitment, coordination and data entry for this trial and members of the CV-BRC PPI group and public who assisted with the review of study documents. Special thanks are also given to perfusion, anaesthetics and nursing teams for their support in the conduct of the trial and to Pierson Ltd and Stefano Marianeschi for their help in providing IPVR training

COMPETING INTERESTS

Pierson Ltd have agreed to provide the injectable tissue valves (BioPulmonic™, Biointegral Surgical Inc.) used in the trial at a discounted rate. Pierson Ltd paid for the expenses associated with Stefano Marianeschi to visit the trial centres and provide IPVR training. Pierson Ltd had no input into the design or conduct of the trial, and will have no access to the data arising from the trial.

FUNDING STATEMENT

The trial is funded by the National Institute for Health Research (NIHR) Bristol Cardiovascular Biomedical Research Centre (Cardiovascular theme) and the British Heart Foundation. The views and opinions expressed herein are those of the authors and do not necessarily reflect those of the NIHR, NHS or the Department of Health. The NIHR and BHF will not be involved in the study conduct, analysis or reporting of the trial.

Availability of data and materials

After study completion and report the data generated from the study will be available from the corresponding author on request. The request must include a specification

For peer review only

of the data requested and justification for that request (i.e. a statement of purpose for which the data are required). The data may not be released unless all CTEU Bristol and Sponsor requirements are fulfilled (e.g. protocol, sample size calculation, valid National Research Ethics Service Committee approval, a collaborative arrangement in place between the Sponsor and the external body).

Additional Figures

Figure 1. Trial Schema showing the recruitment pathway with the number of patients to be recruited, anticipated eligibility and recruitment rates.

LIST OF ABBREVIATIONS

ASD	Atrial septal defect
CMR	Cardiovascular magnetic resonance imaging
CPB	Cardiopulmonary bypass - a technique that temporarily takes over the function of the heart and lungs during surgery, maintaining the circulation of blood and the oxygen content of the body whilst allowing surgeons to access static cardiac tissue.
CHQ	Child Health Questionnaire
CTEU	Clinical Trials and Evaluation Unit
DMSC	Data monitoring and safety committee
EQ-5D	EuroQoL-5D
EQ-5D-Y	EuroQoL-5D - (Youth)
FFD	Fitness for discharge
FFP	Fresh frozen plasma
HAS	Human albumin solution
HRQoL	Health related quality of life
ICU	Intensive care unit
IPVR	Injectable pulmonary valve replacement
NIHR	National Institute for Health Research
PIL	Patient information leaflet

For peer review only

P/GIL	Parent/Guardian information leaflet
PVR	Pulmonary valve replacement (conventional)
RCT	Randomised controlled trial
REC	Research ethics committee
RVOT	Right ventricle outflow tract
SF36	36-Item Short Form Survey
TPVR	Transcatheter pulmonary valve replacement

For peer review only

REFERENCES

1. Sanchez HE, Cornish EM, Shih FC, de Nobrega J, Hassoulas J, Netto J, et al. The surgical treatment of tetralogy of Fallot. Ann Thorac Surg. 1984;37(5):431-6.
2. National Congenital Heart Disease Audit Website 2016 [Available from: https://nicor4.nicor.org.uk/chd/an_paeds.nsf/0/E77F61C7064DA941802580EC0027E335.
3. Berdat PA, Carrel T. Off-pump pulmonary valve replacement with the new Shelhigh Injectable Stented Pulmonic Valve. J Thorac Cardiovasc Surg. 2006;131(5):1192-3.
4. Dittrich S, Gloeckler M, Arnold R, Sarai K, Siepe M, Beyersdorf F, et al. Hybrid pulmonary valve implantation: injection of a self-expanding tissue valve through the main pulmonary artery. Ann Thorac Surg. 2008;85(2):632-4.
5. Schreiber C, Horer J, Vogt M, Fratz S, Kunze M, Galm C, et al. A new treatment option for pulmonary valvar insufficiency: first experiences with implantation of a self-expanding stented valve without use of cardiopulmonary bypass. Eur J Cardiothorac Surg. 2007;31(1):26-30.
6. Boone RH, Webb JG, Horlick E, Benson L, Cao QL, Nadeem N, et al. Transcatheter pulmonary valve implantation using the Edwards SAPIEN transcatheter heart valve. Catheter Cardiovasc Interv. 2010;75(2):286-94.
7. von Wattenwyl R, Arnold R, Siepe M, Beyersdorf F, Schlensak C. Implantation of a catheter-based self-expanding pulmonary valve in congenital heart surgery: results of a pilot study. Eur J Cardiothorac Surg. 2011;40(3):552-6.
8. Marianeschi SM, Santoro F, Ribera E, Catena E, Vignati G, Ghiselli S, et al. Pulmonary valve implantation with the new Shelhigh Injectable Stented Pulmonic Valve. Ann Thorac Surg. 2008;86(5):1466-71; discussion 72.
9. Deorsola L, Abbruzzese PA. Use of oversized injectable valves in growing children for total repair of right ventricular outflow tract anomalies (preliminary results). Tex Heart Inst J. 2014;41(4):373-80.
10. Grohmann J, Hohn R, Fleck T, Stiller B, Neumann E, Sigler M, et al. No-React(R) Injectable BioPulmonic valves re-evaluated: discouraging follow-up results. Interact Cardiovasc Thorac Surg. 2015;21(5):657-65.
11. Chen Q, Turner M, Caputo M, Stoica S, Marianeschi S, Parry A. Pulmonary valve implantation using self-expanding tissue valve without cardiopulmonary bypass reduces operation time and blood product use. J Thorac Cardiovasc Surg. 2013;145(4):1040-5.

12. Boshoff DE, Cools BL, Heying R, Troost E, Kefer J, Budts W, et al. Off-label use of percutaneous pulmonary valved stents in the right ventricular outflow tract: time to rewrite the label? Catheter Cardiovasc Interv. 2013;81(6):987-95.
13. Schievano S, Coats L, Migliavacca F, Norman W, Frigiola A, Deanfield J, et al. Variations in right ventricular outflow tract morphology following repair of congenital heart disease: implications for percutaneous pulmonary valve implantation. J Cardiovasc Magn Reson. 2007;9(4):687-95.

For peer review only

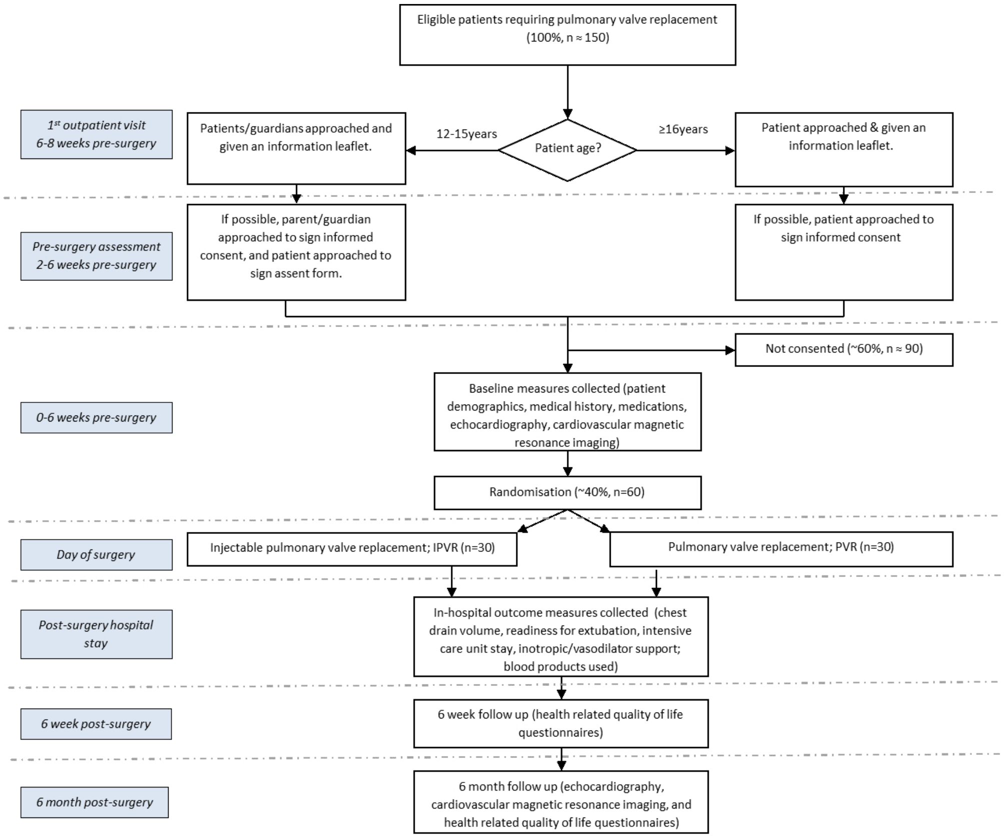

Figure 1. Trial Schema showing the recruitment pathway with the number of patients to be recruited, anticipated eligibility and recruitment rates.

108x90mm (300 x 300 DPI)

SPIRIT
STANDARD PROTOCOL ITEMS: RECOMMENDATIONS FOR INTERVENTIONAL TRIALS

SPIRIT 2013 Checklist: Recommended items to address in a clinical trial protocol and related documents*

Section/item	Item No	Description	Addressed on page number
**Administrative information**			
Title	1	Descriptive title identifying the study design, population, interventions, and, if applicable, trial acronym	______1_______
Trial registration	2a	Trial identifier and registry name. If not yet registered, name of intended registry	______3_______
	2b	All items from the World Health Organization Trial Registration Data Set	_____n/a for protocol paper
Protocol version	3	Date and version identifier	______12_______
Funding	4	Sources and types of financial, material, and other support	______15_______
Roles and responsibilities	5a	Names, affiliations, and roles of protocol contributors	_____1,14____
	5b	Name and contact information for the trial sponsor	_____15_______
	5c	Role of study sponsor and funders, if any, in study design; collection, management, analysis, and interpretation of data; writing of the report; and the decision to submit the report for publication, including whether they will have ultimate authority over any of these activities	__15 and 16____
	5d	Composition, roles, and responsibilities of the coordinating centre, steering committee, endpoint adjudication committee, data management team, and other individuals or groups overseeing the trial, if applicable (see Item 21a for data monitoring committee)	______n/a_____

For peer review only

Introduction

Background and rationale	6a	Description of research question and justification for undertaking the trial, including summary of relevant studies (published and unpublished) examining benefits and harms for each intervention	____4,5_______
	6b	Explanation for choice of comparators	_______5______
Objectives	7	Specific objectives or hypotheses	______5_______
Trial design	8	Description of trial design including type of trial (eg, parallel group, crossover, factorial, single group), allocation ratio, and framework (eg, superiority, equivalence, noninferiority, exploratory)	_______6,7_____

Methods: Participants, interventions, and outcomes

Study setting	9	Description of study settings (eg, community clinic, academic hospital) and list of countries where data will be collected. Reference to where list of study sites can be obtained	_______5______
Eligibility criteria	10	Inclusion and exclusion criteria for participants. If applicable, eligibility criteria for study centres and individuals who will perform the interventions (eg, surgeons, psychotherapists)	_______5_______
Interventions	11a	Interventions for each group with sufficient detail to allow replication, including how and when they will be administered	_______7,8______
	11b	Criteria for discontinuing or modifying allocated interventions for a given trial participant (eg, drug dose change in response to harms, participant request, or improving/worsening disease)	______12_______
	11c	Strategies to improve adherence to intervention protocols, and any procedures for monitoring adherence (eg, drug tablet return, laboratory tests)	________n/a_____
	11d	Relevant concomitant care and interventions that are permitted or prohibited during the trial	_______6_______
Outcomes	12	Primary, secondary, and other outcomes, including the specific measurement variable (eg, systolic blood pressure), analysis metric (eg, change from baseline, final value, time to event), method of aggregation (eg, median, proportion), and time point for each outcome. Explanation of the clinical relevance of chosen efficacy and harm outcomes is strongly recommended	_______8-10_____
Participant timeline	13	Time schedule of enrolment, interventions (including any run-ins and washouts), assessments, and visits for participants. A schematic diagram is highly recommended (see Figure)	______9,10______

For peer review only - http://bmjopen.bmj.com/site/about/guidelines.xhtml

Sample size	14	Estimated number of participants needed to achieve study objectives and how it was determined, including clinical and statistical assumptions supporting any sample size calculations	______10_______
Recruitment	15	Strategies for achieving adequate participant enrolment to reach target sample size	_____12,13_____

Methods: Assignment of interventions (for controlled trials)

Allocation:

Sequence generation	16a	Method of generating the allocation sequence (eg, computer-generated random numbers), and list of any factors for stratification. To reduce predictability of a random sequence, details of any planned restriction (eg, blocking) should be provided in a separate document that is unavailable to those who enrol participants or assign interventions	_____7_______
Allocation concealment mechanism	16b	Mechanism of implementing the allocation sequence (eg, central telephone; sequentially numbered, opaque, sealed envelopes), describing any steps to conceal the sequence until interventions are assigned	_______7______
Implementation	16c	Who will generate the allocation sequence, who will enrol participants, and who will assign participants to interventions	_______7______
Blinding (masking)	17a	Who will be blinded after assignment to interventions (eg, trial participants, care providers, outcome assessors, data aalysts), and how	_____10________
	17b	If blinded, circumstances under which unblinding is permissible, and procedure for revealing a participant's allocated intervention during the trial	NI

Methods: Data collection, management, and analysis

Data collection methods	18a	Plans for assessment and collection of outcome, baseline, and other trial data, including any related processes to promote data quality (eg, duplicate measurements, training of assessors) and a description of study instruments (eg, questionnaires, laboratory tests) along with their reliability and validity, if known. Reference to where data collection forms can be found, if not in the protocol	_____9,10______
	18b	Plans to promote participant retention and complete follow-up, including list of any outcome data to be collected for participants who discontinue or deviate from intervention protocols	11

Data management	19	Plans for data entry, coding, security, and storage, including any related processes to promote data quality (eg, double data entry; range checks for data values). Reference to where details of data management procedures can be found, if not in the protocol	_____N/I______
Statistical methods	20a	Statistical methods for analysing primary and secondary outcomes. Reference to where other details of the statistical analysis plan can be found, if not in the protocol	_____10,11___
	20b	Methods for any additional analyses (eg, subgroup and adjusted analyses)	_____N/A______
	20c	Definition of analysis population relating to protocol non-adherence (eg, as randomised analysis), and any statistical methods to handle missing data (eg, multiple imputation)	_____N/I_____
**Methods: Monitoring**			
Data monitoring	21a	Composition of data monitoring committee (DMC); summary of its role and reporting structure; statement of whether it is independent from the sponsor and competing interests; and reference to where further details about its charter can be found, if not in the protocol. Alternatively, an explanation of why a DMC is not needed	______12_______
	21b	Description of any interim analyses and stopping guidelines, including who will have access to these interim results and make the final decision to terminate the trial	______N/A_____
Harms	22	Plans for collecting, assessing, reporting, and managing solicited and spontaneously reported adverse events and other unintended effects of trial interventions or trial conduct	______N/I_______
Auditing	23	Frequency and procedures for auditing trial conduct, if any, and whether the process will be independent from investigators and the sponsor	______N/I_______
**Ethics and dissemination**			
Research ethics approval	24	Plans for seeking research ethics committee/institutional review board (REC/IRB) approval	_____11______
Protocol amendments	25	Plans for communicating important protocol modifications (eg, changes to eligibility criteria, outcomes, analyses) to relevant parties (eg, investigators, REC/IRBs, trial participants, trial registries, journals, regulators)	_____13________

Consent or assent	26a	Who will obtain informed consent or assent from potential trial participants or authorised surrogates, and how (see Item 32)	______7_____
	26b	Additional consent provisions for collection and use of participant data and biological specimens in ancillary studies, if applicable	____N/A_______
Confidentiality	27	How personal information about potential and enrolled participants will be collected, shared, and maintained in order to protect confidentiality before, during, and after the trial	_____9_______
Declaration of interests	28	Financial and other competing interests for principal investigators for the overall trial and each study site	_____15______
Access to data	29	Statement of who will have access to the final trial dataset, and disclosure of contractual agreements that limit such access for investigators	______16_____
Ancillary and post-trial care	30	Provisions, if any, for ancillary and post-trial care, and for compensation to those who suffer harm from trial participation	______N/A______
Dissemination policy	31a	Plans for investigators and sponsor to communicate trial results to participants, healthcare professionals, the public, and other relevant groups (eg, via publication, reporting in results databases, or other data sharing arrangements), including any publication restrictions	_____3_______
	31b	Authorship eligibility guidelines and any intended use of professional writers	______N/A______
	31c	Plans, if any, for granting public access to the full protocol, participant-level dataset, and statistical code	_____N/A_______
**Appendices**			
Informed consent materials	32	Model consent form and other related documentation given to participants and authorised surrogates	On request
Biological specimens	33	Plans for collection, laboratory evaluation, and storage of biological specimens for genetic or molecular analysis in the current trial and for future use in ancillary studies, if applicable	_____N/A______

*It is strongly recommended that this checklist be read in conjunction with the SPIRIT 2013 Explanation & Elaboration for important clarification on the items. Amendments to the protocol should be tracked and dated. The SPIRIT checklist is copyrighted by the SPIRIT Group under the Creative Commons "Attribution-NonCommercial-NoDerivs 3.0 Unported" license.

For peer review only - http://bmjopen.bmj.com/site/about/guidelines.xhtml
